# Genome-wide association study uncovers a novel QTL allele of *AtS40-3* that affects the sex ratio of cyst nematodes in Arabidopsis

**DOI:** 10.1093/jxb/ery019

**Published:** 2018-01-25

**Authors:** Muhammad Arslan Anwer, Muhammad Shahzad Anjam, Syed Jehangir Shah, M Shamim Hasan, Ali A Naz, Florian M W Grundler, Shahid Siddique

**Affiliations:** 1 Rheinische Friedrich-Wilhelms-University of Bonn, INRES – Molecular Phytomedicine, Karlrobert-Kreiten-Straße, Bonn, Germany; 2 Institute of Molecular Biology and Biotechnology (IMBB), Bahauddin Zakariya University, Multan, Pakistan; 3 Plant Breeding, Institute of Crop Science and Resource Conservation (INRES), University of Bonn, Bonn, Germany

**Keywords:** *Arabidopsis thaliana*, AtS40-3, cyst nematodes, genome-wide association study, plant-parasitic nematodes, PPR proteins, sex ratio, single nucleotide polymorphism, TATA box, quantitative trait locus

## Abstract

Plant-parasitic cyst nematodes are obligate sedentary parasites that infect the roots of a broad range of host plants. Cyst nematodes are sexually dimorphic, but differentiation into male or female is strongly influenced by interactions with the host environment. Female populations typically predominate under favorable conditions, whereas male populations predominate under adverse conditions. Here, we performed a genome-wide association study (GWAS) in an Arabidopsis diversity panel to identify host loci underlying variation in susceptibility to cyst nematode infection. Three different susceptibility parameters were examined, with the aim of providing insights into the infection process, the number of females and males present in the infected plant, and the female-to-male sex ratio. GWAS results suggested that variation in sex ratio is associated with a novel quantitative trait locus allele on chromosome 4. Subsequent candidate genes and functional analyses revealed that a senescence-associated transcription factor, *AtS40-3*, and *PPR* may act in combination to influence nematode sex ratio. A detailed molecular characterization revealed that variation in nematode sex ratio was due to the disturbed common promoter of *AtS40-3* and *PPR* genes. Additionally, single nucleotide polymorphisms in the coding sequence of *AtS40-3* might contribute to the natural variation in nematode sex ratio.

## Teaser

Crop yield losses are influenced by the sex ratio of cyst nematodes. Expression of an Arabidopsis senescence-associated gene (*AtS40-3*) is positively associated with female-biased cyst nematode sex ratio.

## Introduction

Plant parasitic nematodes are obligate biotrophs that develop an intimate parasitic relationship with host plants. These parasites pose a major threat to agriculture worldwide, causing an estimated loss of over US$100 billion annually (Nicol *et al.*, 2011). They are classified into various types based on their feeding habits; however, most of the economic damage is caused by a small group of sedentary endoparasites that includes cyst (*Heterodera* spp. and *Globodera* spp.) and root-knot nematodes (*Meloidogyne* spp.). Among cyst nematodes, *H. schachtii* is a parasite of plant roots that causes severe yield loss to various agronomic crops including sugar beet.

Second-stage infective juveniles (J2) of cyst nematodes enter the roots by piercing epidermal cells and move intracellularly towards the vascular cylinder, where they select a suitable initial syncytial cell (ISC) and establish a long-term feeding site (Wyss and Zunke, 1986; Wyss and Grundler, 1992). ISC selection may take several hours, and once the feeding site has been established, nematodes become sedentary. The ISC expands through local dissolution of cell walls, leading to the formation of a multinucleate, hypertrophied syncytial nurse cell system. The syncytium is the only source of nutrients for the sedentary nematodes (Kyndt *et al.*, 2013; Siddique and Grundler, 2015). A cocktail of nematode-derived secretions that is released into the infected host tissues facilitates nematode migration, host defense modulation, ISC establishment, and syncytium development (Hewezi and Baum, 2013; Quentin *et al.*, 2013; Gardner *et al.*, 2015; Siddique *et al.*, 2015; Holbein *et al.*, 2016; Habash *et al.*, 2017).

Subsequently, nematodes grow and develop into males or females by undergoing three rounds of molting, emerging as J3 larvae, J4 larvae, and adults, respectively. Whereas females remain sedentary throughout their life cycle, male adults leave the syncytium and fertilize females in neighboring roots and plants. Fertilized females perish and their bodies form a cyst containing hundreds of eggs. Cysts protect the eggs for several years until conditions are favorable for hatching. Cyst nematodes are sexually dimorphic, but differentiation into males and females is strongly influenced by the environment. Females feed longer and require 29 times more food as compared with males (Müller *et al.*, 1981). Syncytia induced by females are also 3.7 times larger than those induced by males (Caswell-Chen and Thomason, 1993). Therefore under optimal conditions with an abundant nutrient supply, most juvenile nematodes develop into females (Ali *et al.*, 2013; Elashry *et al.*, 2013; Siddique *et al.*, 2014a, b; Shah *et al.*, 2017). However, more males develop under unfavorable conditions, such as when infestation levels are high, resulting in intraspecific competition (Ellenby, 1954; Koliopanos and Triantaphyllou, 1972; Steele, 1975), or when the parasites encounter poor nutritional conditions inside the hosts (Trudgill, 1967; Johnson and Viglierchio, 1970; Grundler *et al.*, 1991). In the context of crop damage and population dynamics, the sex ratio of cyst nematodes is of fundamental importance. It not only influences the population size in the next generation but also affects the intensity of crop damage, which is usually proportional to number of females. Therefore, understanding the factors that influence the sex of cyst nematodes could be helpful in developing methods to shift the sex ratio of the nematodes towards males.

Because of its small genome size, highly selfing nature, and wide geographical distribution, *Arabidopsis thaliana* is often used as a model organism to understand the genetics and molecular biology of plants. It is naturally distributed across diverse climates and its accessions reveal striking variations in stress responses, making them attractive subjects for studies of stress adaptation (Koornneef *et al.*, 2004). A genome-wide association study (GWAS) is a statistical approach used to examine genetic variants in different individuals that are associated with a trait of interest (Bush and Moore, 2012). This approach has been widely used to investigate the genetic architecture of the human genome, especially as it relates to common variants of disease predisposition. Single nucleotide polymorphisms (SNPs) are single base-pair changes in DNA sequences that are used as markers of a genomic region. Whereas most SNPs have marginal effects on the biological system, some have functional implications such as altering amino acid sequences and changing factors such as mRNA stability and the binding affinity of transcription factors (Hindorff *et al.*, 2009). Although GWASs have been successfully performed in the agronomic crops *Oryza sativa* (rice) and *Hordeum vulgare* (barley), the availability of complete genome sequences of a large number of natural populations makes Arabidopsis an excellent model organism for GWAS (Atwell *et al.*, 2010). GWAS offers several advantages over traditional linkage mapping to dissect quantitative traits at higher resolution because of recombination rate and density of SNP markers across the genome among the natural population of plants. In addition, SNP markers help to calculate a precise population structure for the GWAS, and hence knowledge of genotype pedigree or crosses is not required (Myles *et al.*, 2009). GWASs have successfully been used to map traits such as salt tolerance (Baxter *et al.*, 2010), shade avoidance (Filiault and Maloof, 2012), differing phenotypes (Atwell *et al.*, 2010), flowering traits (Li *et al.*, 2010), and glucosinolate levels (Chan *et al.*, 2011) in Arabidopsis. In the context of plant–nematode interaction, some recent studies have used GWAS to identify novel quantitative trait loci (QTLs) in various crop plants including wheat (Williams *et al.*, 2006; Pariyar *et al.*, 2016a, b), soybean (Zhang *et al.*, 2016), and rice (Dimkpa *et al.*, 2016). In a previous study, 74 different accessions of Arabidopsis were screened for resistance against *H. schachtii* showing a variation in female numbers (Sijmons *et al.*, 1991). However, interactions between cyst nematodes and a range of natural Arabidopsis accessions have not previously been explored with a GWAS approach. In this study, we investigated the susceptibility of a geographically diverse natural population of Arabidopsis to nematode infection using a GWAS approach. Our results suggest that variation in sex ratio is associated with a novel QTL allele on chromosome 4. Subsequent functional analysis revealed that a senescence-associated transcription factor, AtS40-3, and a pentatricopeptide repeat (PPR) protein of the plant influence nematode sex ratio.

## Materials and methods

### Genome-wide association mapping

Arabidopsis plants were grown in Knop medium as described previously (Sijmons *et al.*, 1991). For GWAS, an assembly of geographically distributed natural population of 148 Arabidopsis accessions was ordered from the Nottingham Arabidopsis Stock Centre (NASC; see list of all lines in Supplementary Table S1 at *JXB* online). These accessions are part of a set of 195 natural accessions that were sequenced using the Illumina HiSeq2000 platform by the J. Ecker laboratory at the Salk Institute (USA) as part of the 1001 Genomes Project (NASC ID: N76636). Originally, all 195 accessions were ordered; however, due to germination problems and contamination issues, data from only 148 accessions were collected. Twelve-day-old plants were inoculated with 70–80 J2s of *H. schachtii*. Three experiments were performed, each comprising 50 accessions, each accession with approximately 20 individual plants. A widely used susceptible accession, Col-0, was used as a control in all three experiments. The average number of males and females per plant were counted at 14 days post-inoculation (dpi). Trait values for the number of male and female nematodes across the replicates were normalized to Col-0, whose value was set to 100%. The sex ratio was calculated by dividing the average number of females per plant by the average number of males per plant. Based on the female/male sex ratio, we categorized all genotypes into three susceptibility groups (sex ratio 0–0.5, low susceptibility; sex ratio 0.5–1, moderate susceptibility; sex ratio >1, high susceptibility). Accordingly, 46.6% were low susceptibility, 26% were moderate susceptibility, and 25.4% were high susceptibility. The phenotype data were compared with a 250 K SNP array (V3.6; TAIR9) for GWAS using an accelerated linear mixed-model in a web-based application program, GWAPP (Seren *et al.*, 2012). This model accounts for the correction of confounding from population structure and genetic relatedness in marker trait association analysis. In addition, marker trait associations, which passed the critical threshold of false discovery rate <5%, were considered to be potential candidates. A nominal SNP allele frequency <5% was excluded from the QTL analysis. Finally, two extreme bulks showing lowest and highest susceptibility parameters were selected based on the allelic polymorphism at the associated SNP locus for analysis of candidate genes as well as for functional characterizations.

### Plant growth conditions and nematode infection assays

Transfer DNA (T-DNA)-inserted knockout mutants for selected candidate genes were ordered from the NASC (Supplementary Table S2). All mutants used in this study are in Col-0 background. The homozygosity of SALK mutants was confirmed via PCR using primers shown in Supplementary Table S3. The homozygosity of GABI-Kat lines was confirmed through sulfadiazine resistance screening. Absence of expression of the target gene in the homozygous SALK or GABI-Kat knockout mutants was confirmed through RT-PCR with primers given in Supplementary Table S4. Seeds were sterilized for 4–5 min in 0.7% sodium hypochlorite and subsequently washed four times with sterile water at room temperature. Seedlings were grown in 9 cm Petri dishes under optimized growth conditions at 22 °C with 16 h photoperiod.

Cysts were harvested from the stock culture of mustard plants growing on Knop medium and collected into a cyst-collecting funnel containing sterilized 3 mM ZnCl_2_ to stimulate the hatching. After 1 week, larvae (J2) were harvested and sterilized with 0.05% HgCl_2_ and subsequently washed three times with sterile water at room temperature. Twelve-day-old seedlings were inoculated with 70–80 J2s per plant. The average numbers of males and females were counted at 14 dpi. The average numbers of males and females were used to calculate the sex ratio. All infection assays were repeated a minimum of three times and each experiment consisted of 15–20 individual plants. Average area of syncytia and average area of female nematodes was measured as described previously (Shah *et al.*, 2017). Approximately, 30 syncytia and associated nematodes were measured for each experiment, and each experiment was repeated three times.

### Gene expression analysis by real-time PCR

The roots of 12-day-old uninfected plants from two low susceptibility accessions (Xan-1 and Van-0) and two high susceptibility accessions (Zdr-1 and Kro-0) were harvested, and RNA was extracted using an RNeasy Plant Mini Kit according to the manufacturer’s instructions (Qiagen, Germany). cDNA was synthesized using a High-Capacity cDNA Reverse Transcription Kit (Life Technologies cat. no. 4368814), according to the manufacturer’s instructions. The transcript abundance of targeted genes was analysed using the StepOnePlus Real-Time PCR System (Applied Biosystems). Each sample contained 10 μl of Fast SYBR Green qPCR Master Mix with uracil-DNA, glycosylase, and 6-carboxy-X-rhodamine (Invitrogen), 2 mM MgCl_2_, 0.5 μl of forward and 0.5 μl of reverse primers (10 μM), 2 μl of complementary DNA (cDNA) and water in 20 μl of total reaction volume. Samples were analysed in three biological replicates (independent experiments) and each biological replicate consisted of three technical replicates. To serve as an internal control, *β-tubulin* and *Ubiquitin 5* (*Ubq5*) genes were used. Relative expression was calculated using Pfaffl’s method, by which the expression of the target gene was normalized to internal controls to calculate fold change (Pfaffl, 2001). Primers are listed in Supplementary Table S5.

### Cloning and expression analysis of promoter::GFP

Promoter regions (1500 bp) upstream of ATG of *AtS40-3* from the Xan-1 accession (low susceptibility to nematodes) and Col-0 (moderate susceptibility to nematodes) were amplified from genomic DNA using primers given in Supplementary Table S3 and cloned in a Gateway cloning vector, pDONR207 (Invitrogen), according to the manufacturer’s instructions. The sequence-verified fragments were fused with green fluorescent protein (GFP) in expression vector pMDC107 (Curtis and Grossniklaus, 2003). These constructs were introduced into *Agrobacterium tumefaciens* strain GV3101 for epidermal infiltration of *Nicotiana benthamianna*. Four days after infiltration, RNA was extracted and used to synthesize cDNA as described above. The relative expression of *GFP* with reference to the hygromycin (*hyg*) gene (having constitutive expression under the 35S promoter) was analysed using real-time PCR as described above. The 28 bp promoter sequence from Col-0 was analysed for the presence of *cis*-acting elements using PlantPAN2.0 (http://plantpan2.itps.ncku.edu.tw/;Chow *et al.*, 2015).

## Results

### Arabidopsis accession infection assays

**Fig. 1.**
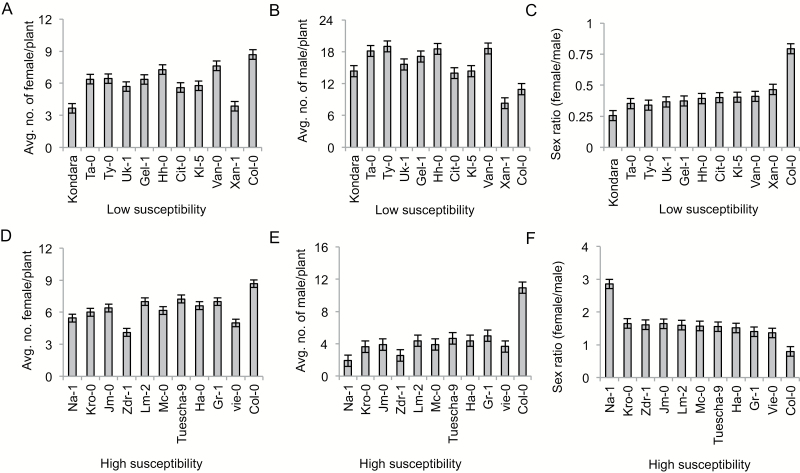
Phenotypic variation in nematode infection in extreme accessions. (A–C) Phenotypic variations in average number of females (A), average number of males (B), and female-to-male sex ratio (C) in low susceptibility accessions. (D–F) Phenotypic variations in average number of females (D), average number of males (E) and female-to-male sex ratio (F) in high susceptibility accessions. Bars represent mean ±SE of 15–20 plants.

To assess the variation in host responses to *Heterodera schachtii*, a natural population of 148 Arabidopsis accessions was analysed via infection assays (Supplementary Fig. S1). In these assays, the average numbers of females and males were recorded at 14 dpi. Based on these data, the female-to-male sex ratio was calculated (see ‘Materials and methods’ for details). The genotypes were categorized into three susceptibility groups (high, moderate, and low susceptibility) (Supplementary Fig. S2). Origin and variation in susceptibility parameters are shown in Table 1 and Fig. 1, respectively, for 20 extreme Arabidopsis accessions responding with low susceptibility (UK-1, Kondara, Xan-1, Van-0, RRS-7, Ta-0, Ty-0, Gel-1, Hh-0, and KI-5) and high susceptibility (Na-1, Vie-0, W1-0, Ha-0, Tuescha-0, Mc-0, Lm-2, Zdr-1, JM-0, and Kr-0) against *H. schachtii*.

**Table 1.** Origin of 20 extreme natural Arabidopsis accessions

No.	Accession	Country	Latitude	Longitude	Phenotype
1	Kondara	Tajikistan	38.48	68.49	Low susceptibility
2	Ta-0	Czech Republic	49.5	14.5	Low susceptibility
3	Ty-0	UK	56.42	-5.23	Low susceptibility
4	Uk-1	Germany	48.03	7.767	Low susceptibility
5	Gel-1	Netherlands	51.01	5.868	Low susceptibility
6	Hh-0	Germany	54.41	8.887	Low susceptibility
7	Cit-0	France	43.37	2.54	Low susceptibility
8	Kl-5	Germany	50.95	6.96	Low susceptibility
9	Van-0	Canada	49.3	-123	Low susceptibility
10	Xan-1	Azerbaijan	48.79	38.65	Low susceptibility
11	Na-1	France	47.5	1.5	High susceptibility
12	Kro-0	Germany	50.07	8.96	High susceptibility
13	Jm-0	Czech Republic	49	15	High susceptibility
14	Zdr-1	Czech Republic	49.38	16.25	High susceptibility
15	Lm-2	France	48	0.05	High susceptibility
16	Mc-0	UK	54.61	-2.3	High susceptibility
17	Tuescha-9	Germany	48.53	9.05	High susceptibility
18	Ha-0	Germany	52.37	9.73	High susceptibility
19	Gr-1	Austria	47	15.5	High susceptibility
20	Wei-0	Switzerland	47.25	8.26	High susceptibility

### GWAS and candidate gene analysis

**Fig. 2.**
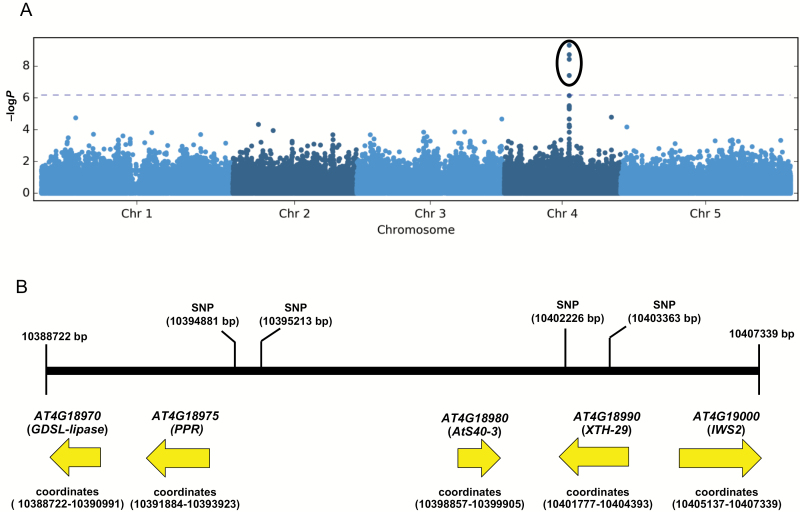
Genetic mapping and isolation of candidate genes. (A) Manhattan plot of marker trait association across the genome. Dashed line indicates Benjamini–Hochberg threshold. (B) Physical map of the genomic region associated to nematode sex ratio indicating significantly associated SNP markers and putative candidate genes.

To perform GWAS, we used GWAPP, a web-based application with a linear mixed model implemented in the program EMMA (http://gwas.gmi.oeaw.ac.at;Seren *et al.*, 2012). SNPs with significance above false discovery rate threshold were considered to be highly associated with the susceptibility parameters of *H. schachtii*. The data analysis revealed a major QTL at chromosome 4 where the SNP positioned at 10402226 bp showed the strongest association with the trait female-to-male sex ratio (Fig. 2A; Supplementary Fig. S3). Moreover, this marker locus revealed decay of linkage disequilibrium with the neighboring SNP markers, which helped us to delimit the targeted genomic region underlying candidate genes associated with the major QTL for sex ratio of nematodes. Using the physical map of this locus, we found five candidate genes at the targeted interval, designated as *GDSL-lipase* (At4g18970), *PPR-protein* (pentatricopeptide repeat-protein; At4g18975), *AtS40-3* (senescence-associated protein; At4g18980), *XTH-29* (xyloglucan endotransglucosylase 29, At4g18990) and *IWS-2* (interacts with SPT6; Atg19000) (Fig. 2B). To examine SNPs in some of our extreme accessions (10 low susceptibility and 10 high susceptibility), we analysed the SNPs in the untranslated, coding, and non-coding regions of our candidate genes (http://signal.salk.edu/atg1001/3.0/gebrowser.php). Out of 20 extreme accessions, sequence data for 18 were available, and although we found polymorphism in single nucleotides and amino acids only with the *AtS40-3* gene, this polymorphism was not consistent (Supplementary Fig. S4).

**Fig. 3.**
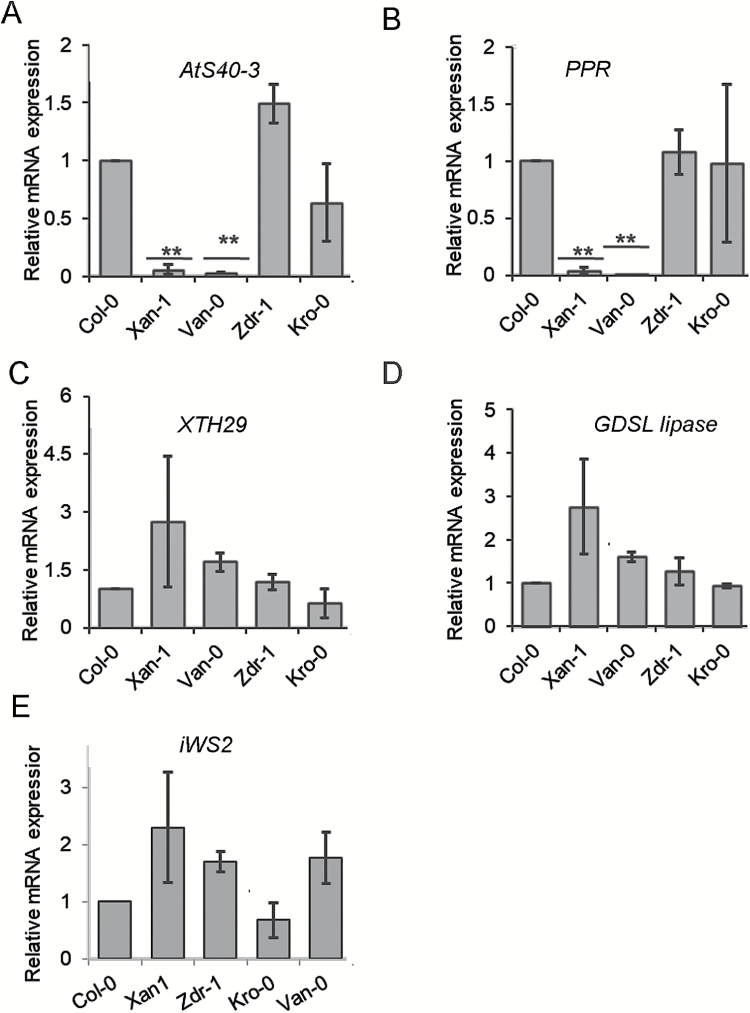
Relative mRNA expression among selected Arabidopsis accessions for *AtS40-3* (A), *PPR* (B), *XTH-29* (C), *GDSL-lipase* (D), and *iWS2* (E). Bars represent the average for three independent experiments ±SE. Data were analysed for significance using Student’s *t*-test; asterisks represent statistically significant difference from corresponding Col-0 (*P*<0.05).

To further test the association with sex ratio of five candidate genes, we measured the expression of candidate genes among four extreme accessions showing low (Xan-1 and Van-0; low susceptibility to nematodes) and high sex ratio (Zdr-1 and Kro-0; high susceptibility to nematodes). The expression of individual genes in the reference accession Col-0 was used as a control. The plants were grown *in vitro* and uninfected roots were collected from 12-day-old plants. RNA was extracted and used to synthesize cDNA. Our qRT-PCR analysis revealed that out of five, expression of two genes, *PPR* (At4g18975) and *AtS40-3* (At4g18980), was strongly down-regulated in low susceptibility accessions as compared with Col-0 (Fig. 3). In comparison, expression of *GDSL-lipase* (At4g18970), *iWS2*, and *XTH29* (At4g18990) was unchanged among all tested accessions suggesting that these genes were not associated with the trait variation. Together, these data suggest that changes in expression level of *PPR* and *AtS40-3* might be correlated with the variation in sex ratio of nematodes in Arabidopsis accessions.

### Sequence variation in promoter region of *AtS40-3* and *PPR*

**Fig. 4.**
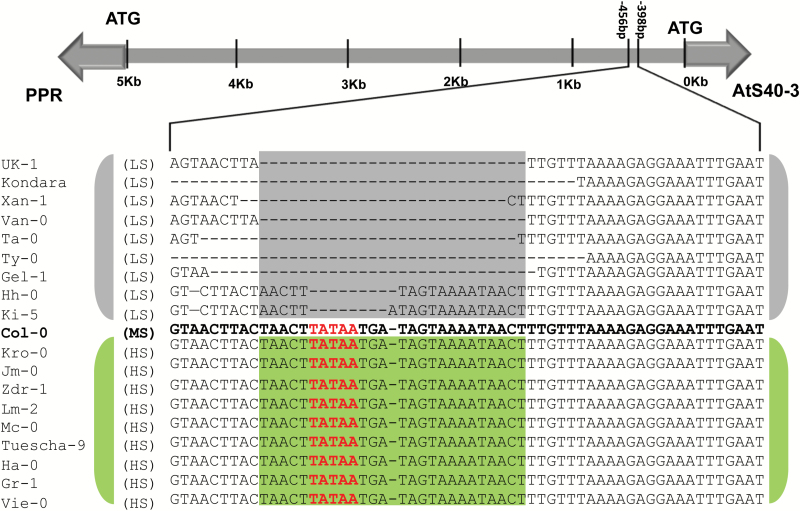
Promoter analysis of *AtS40-3* and *PPR* genes. Promoter region indicating major deletion in the putative bidirectional promoter of *AtS40-3* and *PPR* genes.

*Ats40-3* and *PPR* are divergent genes that are organized head-to-head (in opposite orientations) in a non-overlapping manner. These genes are separated by a distance of approximately 5000 bp and may share a single promoter that acts in a bidirectional manner. Because the expression of *AtS40-3* and *PPR* in roots of low susceptibility accessions was particularly down-regulated, we analysed all candidate genes for sequence variations in their promoter region. Indeed, we observed a considerable polymorphism in the common promoter region among different accessions. Notably, we observed 7–51 bp deletions in the promoter of *AtS40-3* and *PPR* in low susceptibility accessions whereas no such deletion was present in extreme susceptibility accessions (Fig. 4; Supplementary Fig. S5). Based on these data, we hypothesized that this deletion in the promoter region of *AtS40*-3 and *PPR* genes might be one of the reasons for their strong down-regulation in roots tissues in low susceptibility accessions. To further investigate this hypothesis, we performed an *in silico* analysis of the 28-bp promoter region from susceptible line Col-0 for the occurrence of important *cis*-acting elements (Chow *et al.*, 2015). Our analysis revealed the presence of some prominent transcription factor binding sequences including a typical TATA box (Table 2); however, these motifs were deleted in low susceptibility lines. Taken together, these data suggest that deletion of these binding motifs from the upstream region of *AtS40*-*3* and *PPR* genes may be the underlying reason for their strongly decreased expression in low susceptibility lines.

**Table 2.** Promoter sequence (28 bp) from Col-0 with predicted putative *cis*-acting elements

Family	Position	Strand	Similarity score	Hit sequence
AT-Hook	17	+	1	gtaaAATAA
GATA;tify	12	+	1	TGATA
ZF-HD	8	−	1	ATAAT
GATA;tify	13	+	1	GATAG
Trihelix	1	−	0.8	CTAAC
Trihelix	17	−	0.8	GTAAA
Trihelix	22	−	0.8	ATAAC
(Motif sequence only)	17	−	0.8	GTAAA

**Fig. 5.**
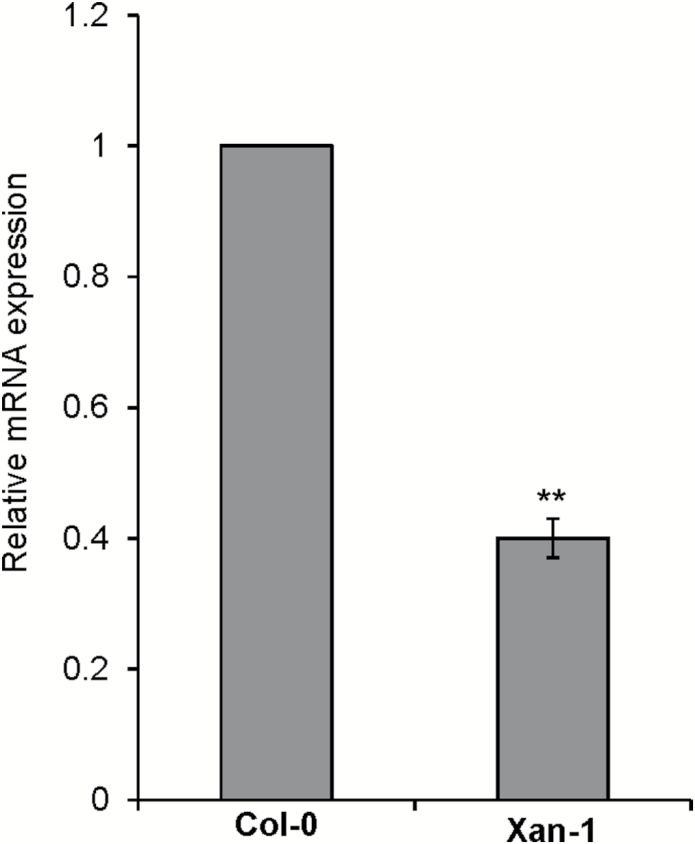
Relative mRNA expression of GFP with *AtS40-3* promoter from Col-0 and Xan-1 accessions. Bars represent the average for three independent experiments ±SE. Data were analysed for significance using Student’s *t*-test; asterisks represent statistically significant difference from corresponding Col-0 (*P*<0.05).

To understand the mechanism that regulates the differential promoter activity for *AtS40-3* between low and high susceptibility lines, the 1500 bp promoter upstream of ATG for *AtS40-3* from the low susceptibility accession Xan-1 (carrying a deletion of 28 nt) and the susceptible accession Col-0 (without deletion) was cloned and used to drive the expression of GFP. The expression of GFP was analysed via a transient expression system in *N. benthamiana* through real-time qPCR. Indeed, we found a significantly reduced expression of GFP driven by the promoter cloned from the Xan-1 accession (low susceptibility to nematodes) compared with that from Col-0 (high susceptibility to nematodes) (Fig. 5).

### Functional characterization of candidate genes

**Fig. 6.**
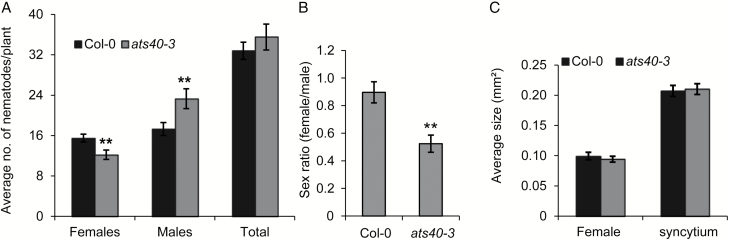
Cyst nematode infection assays in *ats40-3* lines. (A) Average number of females and males per plant present in Col-0 and *ats40-3* lines at 14 dpi. (B) Average sizes of females nematodes and plant syncytia in Col-0 and *ats40-3* lines. Bars represent mean ±SE for three independent experiments. Data were analysed using Student’s *t*-test; asterisks represent statistically significant difference from corresponding Col-0 (*P*<0.05).

To assess which of the five candidate genes underlies the variation in selected genomic region, we obtained the T-DNA insertion loss-of-function mutant lines for all five candidate genes. Homozygous lines were generated and analysed for lack of expression via RT-PCR using specific primers flanking insertion for four candidate genes. However, we were able to get true homozygous loss-of-function mutants for only three genes (*AtS40-3*, *XTH-29*, and *IWS2*). Confirmed knockout lines for these three genes were further evaluated for their responses to nematodes via infection assays (Supplementary Figs S6–S8). We found a significant decrease in number of females and a corresponding increase in number of males per plant in *ats40-3* compared with Col-0. In addition to the number of nematodes, we also measured the female and syncytium sizes but found no significant differences in all tested lines compared with Col-0 (Fig. 6; Supplementary Figs S7 and S8).

## Discussion

In the present study, we explored the natural variation in Arabidopsis to identify host loci underlying variation in susceptibility to cyst nematode infection. We found significant variation in the sex ratio of nematodes, which ranged from 0.25 in Kondara (low susceptibility) to 2.72 in Na-1 (high susceptibility). Similarly, the average number of males ranged from 14.3 in Kondara (low susceptibility) to only 2 in Na-1 (high susceptibility). Twenty extreme associations were then used to repeat the infection assays, thus confirming the robustness of our method. Using marker-trait associations, we found a major locus conferring susceptibility to *H. schachtii* on chromosome 4. The associated SNP revealed higher rates of decay for linkage disequilibrium, which is probably due to genetic diversity of the association panel. There was a higher recombination rate of neighboring SNP markers with the most significant SNP, which facilitated delimiting the QTL region. A further analysis showed that the QTL underlies five candidate genes (*GDSL-lipase*, *PPR*, *AtS40-3*, *XTH-29*, and *IWS2*) in the proximity of the 18 kb region.

### Candidate genes affecting sex ratio of nematodes

We found that out of five candidate genes, expression of two (*AtS40-3* and *PPR*) was strongly reduced in low susceptibility accessions as compared with high susceptibility ones. This reduction in expression of *AtS40-3* and *PPR* strongly suggests polymorphism in their promoter region, which might be the underlying reason for variation in the sex ratio of cyst nematodes. Indeed, analysis of the DNA sequence among the 20 selected extreme accessions (10 high susceptibility and 10 low susceptibility) showed a consistent deletion of 7–51 nucleotides in the common putative promoter of *AtS40-3* and *PPR*. Notably, the same trend of reduced expression was observed when the promoter from a low susceptibility accession was used to drive the expression of *GFP* in *N. benthamiana* leaves.

We propose that expression of *AtS40-3* and *PPR* is positively correlated with susceptibility of Arabidopsis to cyst nematodes. In support of this hypothesis, we found that loss-of-function mutants for *Ats40-3* showed a significant reduction in the susceptibility of plants to cyst nematodes. However, we also found considerable polymorphism in *AtS40-3* among low and high susceptibility accessions. Non-synonymous single nucleotide polymorphism has been shown to play an important role in introducing amino acid changes in corresponding proteins. Polymorphism in the coding region of *AtS40-3* includes the change of arginine to lysine (19), lysine to asparagine (20), asparagine to tyrosine (28), aspartic acid to glutamic acid (39), asparagine to lysine (46) and proline to lysine (63). Although, the introduction of these SNPs in the coding region of *AtS40-3* in extreme accessions was inconsistent, it is still possible that these changes might be additional variants for susceptibility. Determining whether or not these amino acid changes contribute to the susceptibility of Arabidopsis to cyst nematodes is beyond the reach of these studies; however, the fact that changes in expression of *Ats40-3* are positively correlated with susceptibility to nematodes suggests that amino acid substitutions in AtS40-3 in extreme accessions might not be a vital factor for controlling sex ratio in these accessions.

Previous studies revealed that senescence-associated genes (SAGs) are not only expressed during natural senescence but also in response to various stresses such as wounding, darkness, and pathogen infections and in response to treatment of signaling hormones such as ethylene, jasmonate, and abscisic acid (Gan and Amasino, 1997; Lim *et al.*, 2007; Lim and Nam, 2007). *AtS40-3* encodes a nuclear-targeted protein that modulates senescence, and is 3975 bp downstream of the SNP positioned at 10394881. It has been demonstrated that expression of *AtS40-3* is induced upon salicylic acid treatment, upon pathogen attack, and during natural senescence. This induction of *AtS40-3* coincides with a strong upregulation in expression of WRKY-53 transcription factor and senescence-associated gene-12 (*SAG12*). A detailed characterization via expression analyses and T-DNA knockdown mutants showed that *AtS40-3* regulates senescence, either by modulating the expression of the *WRKY-53* or by activating *SAG12* independent of *WRKY53* (Miao *et al.*, 2004).

Previous studies showed that biotrophic pathogens often delay senescence to keep the cells alive. Given the fact that nematodes are biotrophs, and syncytium serves as the sole source of nutrients for nematodes, it is plausible that regulation of senescence plays a role in the maintenance and functioning of syncytium. Indeed, senescence-like symptoms and upregulation of senescence-associated genes have been observed during the resistance response to nematodes in a number of studies (Bleve-Zacheo *et al.*, 1998; Klink *et al.*, 2010). Our data showed that *ats40-3* mutants displayed a significant decrease in numbers of female nematodes and a significant increase in numbers of male nematodes. Based on previous literature and our data, we propose that *AtS40-3* expression positively regulates nematode infection by delaying syncytium senescence, thus ensuring an abundant supply of nutrients to nematodes, which favors the formation of females. On the other hand, knocking out *AtS40*-3 leads to unfavorable conditions inside the syncytium, including the arrival of early senescence, which may support the development of more males. Intriguingly, previous micro-array analysis with microaspirated syncytial content of *H. schachtii* showed that expression of *WRKY53* is strongly up-regulated at 5 and 15 dpi. In contrast, *AtS40-3* does not show any significant change in expression (Szakasits *et al.*, 2009). These observations make it likely that AtS40-3 acts independently of *WRKY53* to regulate infection of *H. schachtii*.

PPR proteins are a large family of plant RNA-binding proteins that mediate several aspects of gene expression through processing, splicing, editing, and translation of mRNAs (Manna, 2015). PPR proteins primarily act in organelles but also in the nucleus. In addition to *Ats40-3*, our data also hints for a positive role of a *PPR* gene (At4g18975) in determining the sex ratio of cyst nematodes. However, the function of this *PPR* gene remained completely obscure until now. Also in the present work, it was not possible to produce a loss-of-function mutant for the *PPR* gene, which precluded further characterization of this gene. Nonetheless considering that formation of a syncytium involves changes in expression of a large number of genes, we speculate that *PPR* positively regulates nematode infection by influencing the expression of genes essential for syncytium formation and nematode development. However, further work is required to characterize the exact role of PPR protein in cyst nematode infection.

## Conclusion

In conclusion, we have demonstrated a significant variation in susceptibility of Arabidopsis to cyst nematode infection. We also identified a novel genomic region on chromosome 4 that underlies the variation in sex ratio of cyst nematodes. Considering the importance of sex ratio in the context of crop damage and population dynamics, this study might pave the way for development of novel breeding strategies against cyst nematode infection.

## Supplementary data

Supplementary data are available at *JXB* online.

Fig S1. Geographical distribution of Arabidopsis accessions all over the world of which around 30% of accessions were collected from Germany.

Fig. S2. Phenotypic variation in female-to-male sex ratio in Arabidopsis accessions.

Fig. S3. Quantile–quantile (Q–Q) plots.

Fig. S4. Amino acid changes in AtS40-3 in extreme accessions.

Fig. S5. Promoter analysis of *AtS40-3* and *PPR* genes in extreme accessions.

Fig. S6. RT-PCR for presence or absence of *AtS40-3* expression in Col-0 or *ats40-3* mutant.

Fig. S7. Cyst nematode infection assays in *xth-29* mutant plants.

Fig. S8. Cyst nematode infection assays in *iws2* mutant plants.

Table S1. Accessions used in this study for different traits

Table S2. T-DNA lines used in study.

Table S3. Primers for genotyping.

Table S4. RT-PCR primers for expression analysis.

Table S5. Primers for qRT-PCR of candidate genes and cloning of promoter fragments.

Supplementary Material
