# Supplementary material for: Genome-wide association study uncovers a novel QTL allele of AtS40-3 that affects the sex ratio of cyst nematodes in Arabidopsis
Source: J Exp Bot. 2018 Jan 25;69(7):1805–14. doi: 10.1093/jxb/ery019 (PMC5889006; doi:10.1093/jxb/ery019)
Supplement: Supplementary Material [file ery019_suppl_supplementary_material.pdf]

Supplementary information

Table S1: Accessions used in this study for different traits

Codes	accessions	Codes	accessions
6929	Kondara	7255	Mh-0
7351	Ty-0	6909	Col-0
8389	Ta-0	6981	Ws-2
7378	Uk-1	6987	Ak-1
7143	Gel-1	9230	Del-10
7169	Hh-0	9169	Kastel-1
7075	Cit-0	5752	Lan-1
7199	Kl-5	8296	Gd-1
6977	Van-0	8374	Rsch-4
7355	Tiv-1	9806	Star-8
6970	Ts-1	6992	Ang-0
7514	RRS-7	6975	Uod-1
6915	Ei-2	5836	B002-3
7282	or-0	6951	Pu2-23
7250	Me-0	7460	Da(1)-12
9065	xan-1	9800	Agu-1
6926	Kin-0	8215	Fei-0
7244	Mnz-0	7520	Lp2-2
6956	Pu2-7	7258	Nw-0
7306	Pog-0	9230	Del-0
6939	Mt-0	8310	Hs-0
7430	Nc-1	8334	Lu-1
7165	Hn-0	6905	Bur-0
7333	sei-0	8420	Kelstrbch-4
7384	Ven-1	7069	Cha-0
7126	Es-0	6900	Bil-5
6968	Tamm-2	6958	Ra-0
7031	Bsch-0	7320	Rou-0
8388	Stw-0	2274	SLSP-30
6922	Gu-o	7352	Te-0
7223	Li-2:1	8213	Pro-0
7524	Rmx-Ao2	6961	Se-0
6990	Amel-1	7283	Ors-1
7404	Wc-1	6929	Kondara
6898	An-1	8233	Dem-4
7330	sapporo-1	7280	Old-1
7250	Me-0	7337	Si-0
7000	Aa-0	9118	Bak-2

7300	Pla-0	6936	Lz-0
7013	Bd-0	7121	En-T
7323	Rubezhnoe1	9169	kastel-1
6919	Ga-0	9127	Yeg-1
9343	dja-1	7523	Pna-17
6979	Wei-0	8325	Lip-0
7382	Utrecht	6978	Wa-1
7244	Mnz-0	9122	Bak-7
7268	Np-0	9179	Ayu-Dag-3
5805	UKID.101	6967	Sq-0
7062	Ca-0	6910	Ct-1
7014	Ba-1	7147	Gie-0
6904	Br-0	7276	Ob-0
8395	Tu-0	7192	Kil-0
6994	Ann-1	6983	Yo-0
7430	Nc-1	6897	Ag-0
7354	Ting-1	8366	Rd-0
7106	Dr-0	6982	Wt-5
7002	Baa-1	8245	Seattle-0
8271	Bu-0	7479	PHW-10
6940	Oy-0	6709	Bg-2
7372	Tscha-1	8378	Sap-0
7344	Sg-1	9805	Sha
6938	Ms-0	8312	Is-0
7166	Hey-1	7411	Wl-0
7015	Bla-1	9806	vie-0
7071	Chat-1	8300	Gr-1
7343	sp-0	7163	Ha-0
8354	Per-1	9806	Tuescha-9
2290	Ste-3	7252	Mc-0
7472	S96	8329	Lm-2
6931	Kz-9	6984	Zdr-1
7424	Jl-3	8313	Jm-0
9805	Lag2-2	7206	Kro-0
7524	Rmx-A02	8343	Na-1
8314	Ka-0		
6945	Nok-3		

Table S2: T-DNA lines used in study.

Nr.	Locus	ID	Source	Annotation
2	At4g18980	N403134	GK-033F02	AtS40-3 senescence regulator protein
3	At4g18990	N661208	SALK_149853C	XTH29
4	At4g19000	N500734	Salk_000734	Transcription factor IWS2

Table S3: Primers for genotyping.

Genes	Forward Primers	Reverse Primers
XTH29	CGCATCATGTCAATAGAAGGG	CGGCAACACAGATTTCATTTC
IWS2	GAGACCGCTCTTGATCAACTG	GAAGCGGTTCTGTAGATTTCG

Table S4: RT-PCR primers for expression analysis.

Genes	Forward Primers	Reverse Primers
XTH29	TGATGGTGATGATGGTATCATGT	CAATAACACACTCCGGTGGA
AtS40-3	TAATGAGATCGAATCCCTTGACTT	TATGGGGTTTGCTTGAGAAGTAAT
IWS2	ATCGAAATTCACAGGGAGGA	ATTTGCCACTTGGAACAAGG

Table S5: Primers for qRT-PCR of candidate genes and cloning of promoter fragments.

Genes	Forward Primers	Reverse Primers
GDSL-like Lipase	TGGGTTTAGGGTGACAAACG	ACAACATTCGCAGCTTCTCC
PPR	TGGGTCTGGACAGAAAGCAC	ATTTGCAACGCCTTAGCTGC
AtS40-3	GAGGGAGAAATGACACCGCC	TCCAAAAAGCCGGTCATCCT
XTH29	AGCCCTCGACGGTTGTAATG	TTCTCGGAACCGTCGCATAG
IWS2	ACACAGCGAGCGAAAGAAAT	AAAATCATCCGCAGGTTCTG
UBQ5 (At3g62250)	GTTAAGCTCGCTGTTCTTCAGT	TCAAGCTTCAACTCCTTCTTTC
β-tubulin 4 (At5g44340)	TTTCCGTACCCTCAAGCTCG	GTGAAGCCTTGCGAATGGGA
GFP6	ACGTGTCTTGTAGTTCCCGT	ACTACCTGTTCCATGGCCAA
Hygromycin	CCGGTCGGCATCTACTCTAT	TTTCGATGATGCAGCTTGGG
pAtS40-3	CTCGCTAATAATTGTCTTTCCATCAATC	ACGGTAGAAAAAAGAAGATCCACAGC

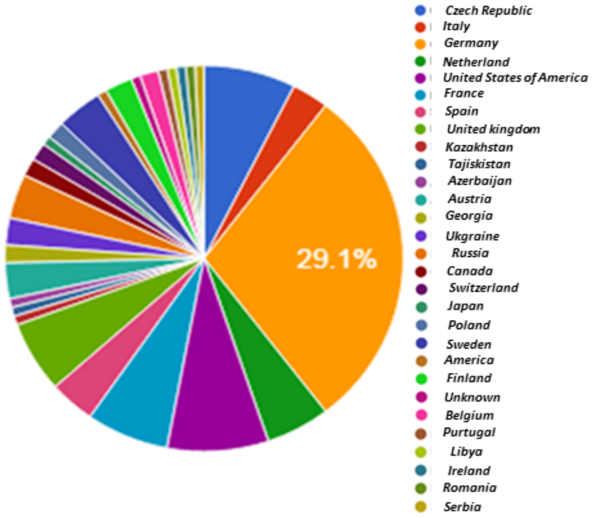

Fig. S1: Geographical distribution of Arabidopsis accessions all over the world of which around 30% accessions were collected from Germany.

Fig S2: Phenotypic variation in female-to-male sex ratio in Arabidopsis accessions.

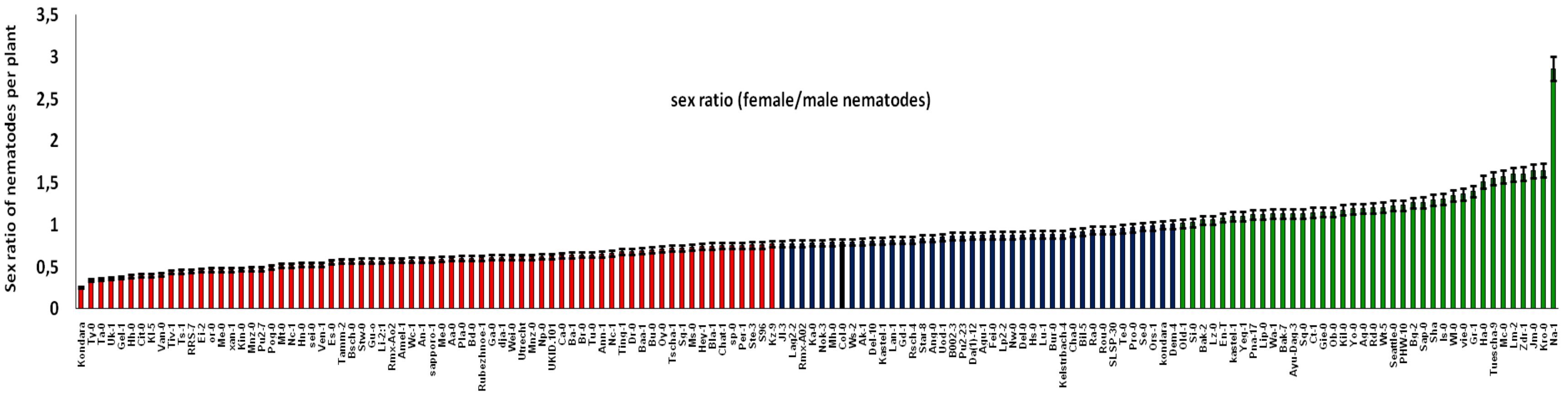

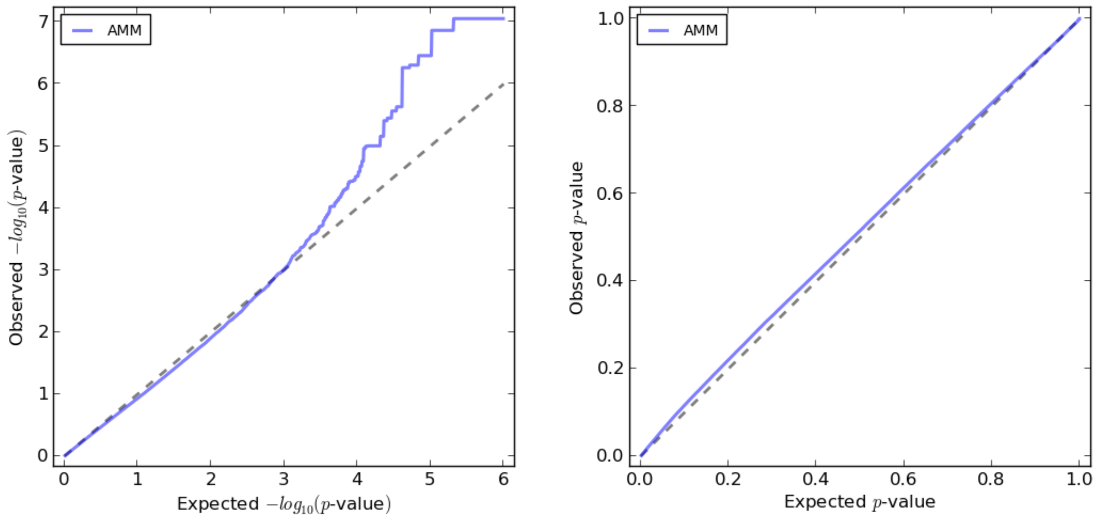

FigS3: Quantile-Quantile (Q–Q) plots. It describes GWAS analysis of variations in Arabidopsis susceptibility in response to cyst nematodes using Accelrated Mix Model.

**Kondara**	MSEEFQESEVIFSDESFTRKDNKISHNNENYERKSTEKEKISSPVRIPSRTTIRYTEEEGEMTPPHVIIEKRRTEAQMAFSFCTLKGRDLSRHRNTVLRMTGFLEV
**Ta-0**	MSEEFQESEVIFSDESFTKNDNKISHNYENYERKSTEKEKISSPVKIPSRTTFRYTEEEGEMKPPHVIIEKRRTEAQMAFSFCTLKGRDLSRRRNTVLRMTGFLEVX
**Ty-0**	MSEEFQESEVIFSDESFTKNDNKISHNYENYERKSTEKEKISSPVKIPSRTTFRYTEEEGEMKPPHVIIEKRRTEAQMAFSFCTLKGRDLSRRRNTVLRMTGFLEVX
**Uk-1**	MSEEFQESEVIFSDESFTKNDNKISHNYENYERKSTEKEKISSPVKIPSRTTFRYTEEEGEMKPPHVIIEKRRTEAQMAFSFCTLKGRDLSRRRNTVLRMTGFLEVX
**Gel-1**	MSEEFQESEVIFSDESFTKNDNKISHNYENYERKSTEKEKISSPVKIPSRTTFRYTEEEGEMKPPHVIIEKRRTEAQMAFSFCTLKGRDLSRRRNTVLRMTGFLEVX
**Hh-0**	MSEEFQESEVIFSDESFTRKDNKISHNNENYERKSTEKDKISSPVRIPSRTTIRYTEEEGEMTPPHVIIEKRRTEAQMAFSFCTLKGRDLSRHRNTVLRMTGFLEVX
**Kl-5**	MSEEFQESEVIFSDESFTRKDNKISHNNENYERKSTEKDKISSPVRIPSRTTIRYTEEEGEMTPPHVIIEKRRTEAQMAFSFCTLKGRDLSRHRNTVLRMTGFLEVX
**Van-0**	MSEEFQESEVIFSDESFTKNDNKISHNYENYERKSTEKEKISSPVKIPSRTTFRYTEEEGEMKPPHVIIEKRRTEAQMAFSFCTLKGRDLSRRRNTVLRMTGFLEVX
**Xan-1**	MSEEFQESEVIFSDESFTKNDNKISHNYENYERKSTEKEKISSPVKIPSRTTFRYTEEEGEMKPPHVIIEKRRTEAQMAFSFCTLKGRDLSRRRNTVLRMTGFLEVX

**Kro-0**	MSEEFQESEVIFSDESFTRKDNKISHNNENYERKSTEKDKISSPVRIPSRTTIRYTEEEGEMTPPHVIIEKRRTEAQMAFSFCTLKGRDLSRHRNTVLRMTGFLEVX
**Jm-0**	MSEEFQESEVIFSDESFTRKDNKISHNNENYERKSTEKDKISSPVRIPSRTTIRYTEEEGEMTPPHVIIEKRRTEAQMAFSFCTLKGRDLSRHRNTVLRMTGFLEVX
**Zdr-1**	MSEEFQESEVIFSDESFTRKDNKISHNNENYERKSTEKDKISSPVRIPSRTTIRYTEEEGEMTPPHVIIEKRRTEAQMAFSFCTLKGRDLSRHRNTVLRMTGFLEVX
**Lm-2**	MSEEFQESEVIFSDESFTKNDNKISHNYENYERKSTEKEKISSPVKIPSRTTFRYTEEEGEMKPPHVIIEKRRTEAQMAFSFCTLKGRDLSRRRNTVLRMTGFLEVX
**Mc-0**	MSEEFQESEVIFSDESFTRKDNKISHNNENYERKSTEKDKISSPVRIPSRTTIRYTEEEGEMTPPHVIIEKRRTEAQMAFSFCTLKGRDLSRHRNTVLRMTGFLEVX
**Tuescha-9**	MSEEFQESEVIFSDESFTRKDNKISHNNENYERKSTEKDKISSPVRIPSRTTIRYTEEEGEMTPPHVIIEKRRTEAQMAFSFCTLKGRDLSRHRNTVLRMTGFLEVX
**Ha-0**	MSEEFQESEVIFSDESFTRKDNKISHNNENYERKSTEKDKISSPVRIPSRTTIRYTEEEGEMTPPHVIIEKRRTEAQMAFSFCTLKGRDLSRHRNTVLRMTGFLEVX
**Gr-1**	MSEEFQESEVIFSDESFTRKDNKISHNNENYERKSTEKDKISSPVRIPSRTTIRYTEEEGEMTPPHVIIEKRRTEAQMAFSFCTLKGRDLSRHRNTVLRMTGFLEVX
**Vie-0**	MSEEFQESEVIFSDESFTKNDNKISHNYENYERKSTEKEKISSPVKIPSRTTFRYTEEEGEMKPPHVIIEKRRTEAQMAFSFCTLKGRDLSRRRNTVLRMTGFLEVX

Fig S4: Amino acid changes in AtS40-3 in extreme accessions.

```
Uk_1          tcatttcttttattttgttattgtttgtatatctcgctcgatcttacattactttgtttt
Kondara       tcatttcttttctttagttattgtttgtatatctcgctcgatcttacattactttgtttt
Xan-1         tcatttcttttattttgttattgtttgtatatctcgctcgatcttacattactttgtttt
Van_0         tcatttcttttattttgttattgtttgtatatctcgctcgatcttacattactttgtttt
Ta_0          tcatttcttttctttagttattgtttgtatatctcgctcgatcttacattactttgtttt
Ty_0          tcatttcttttattttgttattgtttgtatatctcgctcgatcttacattactttgtttt
Gel_1         tcatttcttttattttgttattgtttgtatatctcgctcgatcttacattactttgtttt
Hh_0          tcatttcttttctttagttattgtttgtatatctcgctcgatcttacattactttgtttt
Kl-5          tcatttcttttctttagttattgtttgtatatctcgctcgatcttacattactttgtttt
Kro_0         tcatttcttttctttagttattgtttgtatatctcgctcgatcttacattactttgtttt
Jm_0          tcatttcttttctttagttattgtttgtatatctcgctcgatcttacattactttgtttt
Zdr_1         tcatttcttttctttagttattgtttgtatatctcgctcgatcttacattactttgtttt
Lm-2          tcatttcttttctttagttattgtttgtatatctcgctcgatcttacattactttgtttt
Mc_0          tcatttcttttctttagttattgtttgtatatctcgctcgatcttacattactttgtttt
Tuescha-9     tcatttcttttctttagttattgtttgtatatctcgctcgatcttacattactttgtttt
Ha_0          tcatttcttttctttagttattgtttgtatatctcgctcgatcttacattactttgtttt
Gr_1          tcatttcttttctttagttattgtttgtatatctcgctcgatcttacattactttgtttt
Vie-0         tcatttcttttctttagttattgtttgtatatctcgctcgatcttacattactttgtttt
              *********** *** ********************************************
Uk_1          cttgagcatcgatcaatatcaatatgcaattaaaatatatcaagattaaaaataaaaaga
Kondara       cttgagcatcgatcaatatcaatatgcaattaaaatatatcaagattaaaaataaaaaga
Xan-1         cttgagcatcgatcaatatcaatttgcaattaaaatatatcaagattaaaaataaaaaga
Van_0         cttgagcatcgatcaatatcaatatgcaattaaaatatatcaagattaaaaataaaaaga
Ta_0          cttgagcatcgatcaatatcaatatgcaattaaaatatatcaagattaaaaataaaaaga
Ty_0          cttgagcatcgatcaatatcaatatgcaattaaaatatatcaagattaaaaataaaaaga
Gel_1         cttgagcatcgatcaatatcaatatgcaattaaaatatatcaagattaaaaataaaaaga
Hh_0          cttgagcatcgatcaatatcaatatgcaattaaaatatatcaagattaaaaataaaaaga
Kl-5          cttgagcatcgatcaatatcaatatgcaattaaaatatatcaagattaaaaataaaaaga
Kro_0         cttgagcatcgatcaatatcaatatgcaattaaaatatatcaagattaaaaataaaaaga
Jm_0          cttgagcatcgatcaatatcaatatgcaattaaaatatatcaagattaaaaataaaaaga
Zdr_1         cttgagcatcgatcaatatcaatatgcaattaaaatatatcaagattaaaaataaaaaga
Lm-2          cttgagcatcgatcaatatcaatatgcaattaaaatatatcaagattaaaaataaaaaga
Mc-0          cttgagcatcgatcaatatcaatatgcaattaaaatatatcaagattaaaaataaaaaga
Tuescha-9     cttgagcatcgatcaatatcaatatgcaattaaaatatatcaagattaaaaataaaaaga
Ha_0          cttgagcatcgatcaatatcaatatgcaattaaaatatatcaagattaaaaataaaaaga
Gr_1          cttgagcatcgatcaatatcaatatgcaattaaaatatatcaagattaaaaataaaaaga
Vie-0         cttgagcatcgatcaatatcaatatgcaattaaaatatatcaagattaaaaataaaaaga
              *********************** ************************************
Uk_1          gtgttttggacaaaagaaactcccgaagaagttagacattaaaagctaataatagacgca
Kondara       gtgttttggacaaaagaaactcccgaagaagttagacattaaaagctaataatagacgca
Xan-1         gtgttttggacaaaagaaactcccgaagaagttagacattaaaagctaataatagacgca
Van_0         gtgttttggacaaaagaaactcccgaagaagttagacattaaaagctaataatagacgca
Ta_0          gtgttttggacaaaagaaactcccgaagaagttagacattaaaagctaataatagacgca
Ty_0          gtgttttggacaaaagaaactcccgaagaagttagacattaaaagctaataatagacgca
Gel_1         gtgttttggacaaaagaaactcccgaagaagttagacattaaaagctaataatagacgca
Hh_0          gtgttttggacaaaagaaactcccgaagaagttagacattaaaagctaataatagacgca
Kl-5          gtgttttggacaaaagaaactcccgaagaagttagacattaaaagctaataatagacgca
Kro_0         gtgttttggacaaaagaaactcccgaagaagttagacattaaaagctaataatagacgca
Jm_0          gtgttttggacaaaagaaactcccgaagaagttagacattaaaagctaataatagacgca
Zdr_1         gtgttttggacaaaagaaactcccgaagaagttagacattaaaagctaataatagacgca
Lm-2          gtgttttggacaaaagaaactcccgaagaagttagacattaaaagctaataatagacgca
Mc_0          gtgttttggacaaaagaaactcccgaagaagttagacattaaaagctaataatagacgca
Tuescha-9     gtgttttggacaaaagaaactcccgaagaagttagacagtaaaagctaataatagacgca
Ha_0          gtgttttggacaaaagaaactcccgaagaagttagacattaaaagctaataatagacgca
Gr_1          gtgttttggacaaaagaaactcccgaagaagttagacattaaaagctaataatagacgca
Vie-0         gtgttttggacaaaagaaactcccgaagaagttagacattaaaagctaataatagacgca
              ************************************** *********************
Uk_1          atggaaaaaccaatcttttaagggttttaaagtgtttaaccatgggaactcaaactttga
```

```
Kondara        atggaaaaaccaatcttttaaggctttaa--gtatttaaccatgggaactcaatctttga
Xan-1          atggaaaaaccaatcttttaaggtttt--aagtgtttaaccatgggaactcaaactttga
Van_0          atggaaaaaccaatcttttaagggttt--aagtatttaaccatgggaactcaaactttga
Ta_0           atggaaaaacccatcttttaa--------------ttaaccatgggaactcaatctttga
Ty_0           atggaaaaaccaatcttttaaggctttaagtat--ttaaccatgg---------------
Gel_1          atggaaaaaccaatcttttaagggtttaa--gtatttaaccatgggaactcaaactttga
Hh_0           atggaaaaaccaatcttttaaggctttaa--gtatttaaccatgggaactcaatctttga
Kl-5           atggaaaaaccaatcttttaaggctttaa--gtatttaaccatgggaactcaatctttga
Kro_0          atggaaaaacccatcttttaaggctttaa--gtatttaaccatgggaactcaatctttga
Jm_0           atggaaaaacccatcttttaaggctttaa--gtatttaaccatgggaactcaatctttga
Zdr_1          atggaaaaacccatcttttaaggctttaa--gtatttaaccatgggaactcaatctttga
Lm-2           atggaaaaacccatcttttaaggctttaa--gtatttaaccatgggaactcaatctttga
Mc_0           atggaaaaacccatcttttaaggctttaa--gtatttaaccatgggaactcaatctttga
Tuescha-9      atggaaaaaccaatcttttaaggctttaa--gtatttaaccatgggaactcaatctttga
Ha_0           atggaaaaaccaatcttttaaggctttaa--gtatttaaccatgggaactcaatctttga
Gr_1           atggaaaaaccaatcttttaaggctttaa--gtatttaaccatgggaactcaatctttga
Vie-0          atggaaaaaccaatcttttaaggtttta—-agtgtttaaccatgggaactcaaactttga
               *********** *********              **********

Uk_1           tttatcccccttttttttcttgtccgaaagcaacaaatcaatgatcagccgttaattaac
Kondara        tttatccc--cttttttttcttgtccgaaagcaacaaatcaatgatcagccgttaattaac
Xan-1          tttatcccc--tttttttcttgtccgaaagcaacaaatcaatgatcagccgttaattaac
Van_0          tttatcccccttttttttcttgtccgaaagcaacaaatcaatgatcagccgttaattaac
Ta_0           tttatccc--ctttttttcttgtccgaaagcaacaaatcaatgatcagccgttaattaac
Ty_0           ---ttatccccttttttttcttgtccgaaagcaacaaatcaatgatcagccgttaattaac
Gel_1          tttatcccccttttttttcttgtccgaaagcaacaaatcaatgatcagccgttaattaac
Hh_0           tttatccc--c-tttttcttgtccgaaagcaacaaatcaatgatcagccgttaattgac
Kl-5           tttatccc--c-tttttcttgtccgaaagcaacaaatcaatgatcagccgttaattgac
Kro_0          tttatccc--ctttttttcttgtccgaaagcaacaaatcaatgatcagccgttaattaac
Jm_0           tttatccc--ctttttttcttgtccgaaagcaacaaatcaatgatcagccgttaattaac
Zdr_1          tttatccc--ctttttttcttgtccgaaagcaacaaatcaatgatcagccgttaattaac
Lm-2           tttatccc--ctttttttcttgtccgaaagcaacaaatcaatgatcagccgttaattaac
Mc_0           tttatccc--ctttttttcttgtccgaaagcaacaaatcaatgatcagccgttaattaac
Tuescha-9      tttctccc--c-tttttcttgtccgaaagcaacaaatcaatgatcagccgttaattgac
Ha_0           tttatccc--ctttttttcttgtccgaaagcaacaaatcaatgatcagccgttaattaac
Gr_1           tttatccc--ctttttttcttgtccgaaagcaacaaatcaatgatcagccgttaattaac
Vie-0          tttatccc--ctttttttcttgtccgaaagcaacaaatcaatgatcagccgttaattaac
                   *  *    ********************************************* **

Uk_1           gccgccacgtaatcgcgcgaaggagaacgacggcagttatagattaaacaaacacgtgtc
Kondara        gccgccacgtaatcgcgcgaaggagaacgacggcagttatagattaaacaaacacgtgtc
Xan-1          gccgccacgtaatcgcgcgaaggagaacgacggcagttatagattaaacaaacacgtgtc
Van_0          gccgccacgtaatcgcgcgaaggagaacgacggcagttatagattaaacaaacacgtgtc
Ta_0           gccgccacgtaatcgcgcgaaggagaacgacggcagttatagattaaacaaacacgtgtc
Ty_0           gccgccacgtaatcgcgcgaaggagaacgacggcagttatagattaaacaaacacgtgtc
Gel_1          gccgccacgtaatcgcgcgaaggagaacgacggcagttatagattaaacaaacacgtgtc
Hh_0           gccgccacgtaatcgcgcgaaggagaacgacggcagttatagattaaacaaacacgtgtc
Kl-5           gccgccacgtaatcgcgcgaaggagaacgacggcagttatagattaaacaaacacgtgtc
Kro_0          gccgccacgtaatcgcgcgaaggagaacgacggcagttatagattaaacaaacacgtggc
Jm_0           gccgccacgtaatcgcgcgaaggagaacgacggcagttatagattaaacaaacacgtggc
Zdr_1          gccgccacgtaatcgcgcgaaggagaacgacggcagttatagattaaacaaacacgtggc
Lm-2           gccgccacgtaatcgcgcgaaggagaacgacggcagttatagattaaacaaacacgtggc
Mc_0           gccgccacgtaatcgcgcgaaggagaacgacggcagttatagattaaacaaacacgtggc
Tuescha-9      gccgccacgtaatcgcgcgaaggagaacgacggcagttatagattaaacaaacacgtgtc
Ha_0           gccgccacgtaatcgcgcgaaggagaacgacggcagttatagattaaacaaacacgtggc
Gr_1           gccgccacgtaatcgcgcgaaggagaacgacggcagttatagattaaacaaacacgtggc
Vie-0          gccgccacgtaatcgcgcgaaggagaacgacggcagttatagattaaacaaacacgtgtc
               ********************************************************** *

Uk_1           accggcacacgctagacggtagctttgtgatgttaactttggctgacgttaagctagttt
Kondara        accggcacacgctagacggtagctttgtgatgttaactttggctgacgttaagctagttt
Xan-1          accggcacacgctagacggtagctttgtgatgttaactttggctgacgttaagctagttt
Van_0          accggcacacgctagacggtagctttgtgatgttaactttggctgacgttaagctagttt
Ta_0           accggcacacgctagacggtagctttgtgatgttaactttggctgacgttaagctagttt
Ty_0           accggcacacgctagacggtagctttgtgatgttaactttggctgacgttaagctagttt
Gel_1          accggcacacgctagacggtagctttgtgatgttaactttggctgacgttaagctagttt
```

```
Hh_0            accggcacacgctagacggaagctttgtgacgttaactttggctgacgttaagctagttt
Kl-5            accggcacacgctagacggaagctttgtgacgttaactttggctgacgttaagctagttt
Kro_0           accggcacacgctagacggaagctttgtgacattaactttggctgacgttaagctagttt
Jm_0            accggcacacgctagacggaagctttgtgacattaactttggctgacgttaagctagttt
Zdr_1           accggcacacgctagacggaagctttgtgacattaactttggctgacgttaagctagttt
Lm-2            accggcacacgctagacggaagctttgtgacattaactttggctgacgttaagctagttt
Mc_0            accggcacacgctagacggaagctttgtgacattaactttggctgacgttaagctagttt
Tuescha-9       accggcacacgctagacggaagctttgtgacgttaactttggctgacgttaagctagttt
Ha_0            accggcacacgctagacggaagctttgtgacattaactttggctgacgttaagctagttt
Gr_1            accggcacacgctagacggaagctttgtgacattaactttggctgacgttaagctagttt
Vie-0           accggcacacgctagacggtagctttgtgatgttaactttggctgacgttaagctagttt
                ******************* **********  ****************************

Uk_1            tcgacacgacacgtgttaacgcacacgtgtgggttctcatgttt-tttgtgattattcat
Kondara         tcgacacgacacgtgttaacgcacacgtgtgggttctcgtgttt-tttgtgattattcat
Xan-1           tcgacacgacacgtgttaacgcacacgtgtgggttctcatgttt-tttgtgattattcat
Van_0           tcgacacgacacgtgttaacgcacacgtgtgggttctcatgttt-tttgtgattattcat
Ta_0            tcgacacgacacgtgttaacgcacacgtgtgggttctcatgttt-tttgtgattattcat
Ty_0            tcgacacgacacgtgttaacgcacacgtgtgggttctcatgttt-tttgtaattattcat
Gel_1           tcgacacgacacgtgttaacgcacacgtgtgggttctcatgttt-tttgtgattattcat
Hh_0            tcgacacgacacgtgttagcgcacacgtgtgggttctcatgatttttttgtgattattcat
Kl-5            tcgacacgacacgtgttagcgcacacgtgtgggttctcatgatttttttgtgattattcat
Kro_0           tcgacacgacacgtgttagcgcacacgtgtgggttctcatgttt-tttgtgataattcat
Jm_0            tcgacacgacacgtgttagcgcacacgtgtgggttctcatgttt-tttgtgataattcat
Zdr_1           tcgacacgacacgtgttagcgcacacgtgtgggttctcatgttt-tttgtgataattcat
Lm-2            tcgacacgacacgtgttagcgcacacgtgtgggttctcatgttt-tttgtgataattcat
Mc_0            tcgacacgacacgtgttagcgcacacgtgtgggttctcatgttt-tttgtgataattcat
Tuescha-9       tcgacacgacacgtgttagcgcacacgtgtgggttctcatgttt-tttgtgagtattcat
Ha_0            tcgacacgacacgtgttagcgcacacgtgtgggttctcatgttt-tttgtgataattcat
Gr_1            tcgacacgacacgtgttagcgcacacgtgtgggttctcatgttt-tttgtgataattcat
Vie-0           tcgacacgacacgtgttaacgcacacgtgtgggttctcatgttt-tttgtgattattcat
                ****************** ******************* ** ** ***** *  ******

Uk_1            attttagcgattataagtcgtagagtttgcaaactgcaatcattgaagaaatttcgtcgt
Gel_1           atttaagcgattataagtcgtagagtttgcaaattgcaatcattgaagaatttcgttgta
Kondara         atttaagcgattataagtcgtagagtttgcaaattgcaatcattgaagaa----------
Xan-1           attttagcgattataagtcgtagagtttgcaaattgcaatcattgaagaatt-tcgtcgt
Van_0           attttagcgattataagtcgtagagtttgcaaactgcaatcattgaagaaatttcgtcgt
Ta_0            attttagcgattataagtcgtagagtttgcaaactgcaatcattgaagaaatttcgttgt
Ty_0            attttagcgattataagtcgtagagtttgcaaactgcaatcattgaag------------
Hh_0            atttaagcgattataagtcgtagagtttgcaaactgcaatcattgaagaatttcgttgta
Kl-5            atttaagcgattataagtcgtagagtttgcaaactgcaatcattgaagaatttcgttgta
Kro_0           atttaagcgattataagtcgtagagtttgcaaactgcaatcattgaagaatttcgttgta
Jm_0            atttaagcgattataagtcgtagagtttgcaaactgcaatcattgaagaatttcgttgta
Zdr_1           atttaagcgattataagtcgtagagtttgcaaactgcaatcattgaagaatttcgttgta
Lm-2            atttaagcgattataagtcgtagagtttgcaaactgcaatcattgaagaatttcgttgta
Mc_0            atttaagcgattataagtcgtagagtttgcaaactgcaatcattgaagaatttcgttgta
Tuescha-9       atttaagcgattataagtcgtagagtttgcaaattgcaatcattgaagaatttcgttgta
Ha_0            atttaagcgattataagtcgtagagtttgcaaactgcaatcattgaagaatttcgttgta
Gr_1            atttaagcgattataagtcgtagagtttgcaaactgcaatcattgaagaatttcgttgta
Vie-0           attttagcgattataagtcgtagagtttgcaaattgcaatcattgaagaatttcgtt---
                **** **************************** **************

Uk_1            agtaactta---------------------------ttgtttaaaagaggaaatttgaat
Kondara         -----------------------------------------taaaagaggaaatttgaat
Xan-1           agtaact---------------------------ctttgtttaaaagaggaaatttgaat
Van_0           agtaactta---------------------------ttgtttaaaagaggaaatttgaat
Ta_0            agt--------------------------------tttgtttaaaagaggaaatttgaat
Ty_0            ------------------------------------------aaaagaggaaatttgaat
Gel_1           gtaa---------------------------------tgtttaaaagaggaaatttgaat
Hh_0            gt—cttactaactt---------tagtaaaataactttgtttaaaagaggaaatttgaat
Kl-5            gt—cttactaactt--------atagtaaaataactttgtttaaaagaggaaatttgaat
Kro_0           gtaacttactaacttataatga-tagtaaaataactttgtttaaaagaggaaatttgaat
```

```
Jm_0            gtaacttactaacttataatga-tagtaaaataactttgtttaaaagaggaaatttgaat
Zdr_1           gtaacttactaacttataatga-tagtaaaataactttgtttaaaagaggaaatttgaat
Lm-2            gtaacttactaacttataatga-tagtaaaataactttgtttaaaagaggaaatttgaat
Mc_0            gtaacttactaacttataatga-tagtaaaataactttgtttaaaagaggaaatttgaat
Tuescha-9       gtaacttactaacttataatga-tagtaaaataactttgttt-aaagaggaaatttgaat
Ha_0            gtaacttactaacttataatgaatagtaaaataactttgtttaaaagaggaaatttgaat
Gr_1            gtaacttactaacttataatga-tagtaaaataactttgtttaaaagaggaaatttgaat
Vie-0           gtaacttactaacttataatga-tagtaaaataactttgtttaaaagaggaaatttgaat
                              *****************

Uk_1            gaaaagaattgtgatgttactaattacgtacacatacgcaattatgataaggcaaaatga
Kondara         gaaaagaattgtgatgttactaattacgtacacatacgcaattatgataaggcaaaatga
Xan-1           gaaaagaattgtgatgttactaattacgtacacatacgcaattatgataaggcaaaatga
Van_0           gaaaagaattgtgatgttactaattacgtacacatacgcaattatgataaggcaaaatga
Ta_0            gaaaagaattgtgatgttactaattacgtacacatacgcaattatgataaggcaaaatga
Ty_0            gaaaagaattgtgatgttactaattacgtacacatacgcaattatgataaggcaaaatga
Gel_1           gaaaagaattgtgatgttactaattacgtacacatacgcaattatgataaggcaaaatga
Hh_0            gaaaagaattgtgatgttactaattacgtacacctacgcaattatgataaggcaaaatga
Kl-5            gaaaagaattgtgatgttactaattacgtacacctacgcaattatgataaggcaaaatga
Kro_0           gaaaagaattgtgatgttactaattacgtacacatacgcaattattataaggcaaaatga
Jm_0            gaaaagaattgtgatgttactaattacgtacacatacgcaattattataaggcaaaatga
Zdr_1           gaaaagaattgtgatgttactaattacgtacacatacgcaattattataaggcaaaatga
Lm-2            gaaaagaattgtgatgttactaattacgtacacatacgcaattattataaggcaaaatga
Mc_0            gaaaagaattgtgatgttactaattacgtacacatacgcaattattataaggcaaaatga
Tuescha-9       gaaaagaattgtgatgttactaattacgtacacctacgcaattatgataaggcaaaatga
Ha_0            gaaaagaattgtgatgttactaattacgtacacatacgcaattattataaggcaaaatga
Gr_1            gaaaagaattgtgatgttactaattacgtacacatacgcaattattataaggcaaaatga
Vie-0           gaaaagaattgtgatgttactaattacgtacacatacgcaattatgataaggcaaaatga
                ********************************* *********** **************

Uk_1            tgtgaaagtgtatataaagacttgtgtagttcacgataatcacatccttaatgattaatt
Kondara         tgtgaaagtgtatataaagacttgtgtagttcacgataatcacatccttaatgattaatt
Xan-1           tgtgaaagtgtatataaagacttgtgtagttcacgataatcacatccttaacgattaatt
Van_0           tgtgaaagtgtatataaagacttgtgtagttcacgataatcacatccttaatgattaatt
Ta_0            tgtgaaagtgtatataaagacttgtgtagttcacgataatcacatccttaatgattaatt
Ty_0            tgtgaaagtgtatataaagacttgtgtagttcacgataatcacatccttaatgattaatt
Gel_1           tgtgaaagtgtatataaagacttgtgtagttcacgataatcacatccttaatgattaatt
Hh_0            tgtgaaagtgtatataaagacttgtgtagttcacgataatcacatccttaatgattaatt
Kl-5            tgtgaaagtgtatataaagacttgtgtagttcacgataatcacatccttaatgattaatt
Kro_0           tgtgaaagtgtaaataaagacttgtgtagttcacgataatcacatccttaatgattaatt
Jm_0            tgtgaaagtgtaaataaagacttgtgtagttcacgataatcacatccttaatgattaatt
Zdr_1           tgtgaaagtgtaaataaagacttgtgtagttcacgataatcacatccttaatgattaatt
Lm-2            tgtgaaagtgtaaataaagacttgtgtagttcacgataatcacatccttaatgattaatt
Mc_0            tgtgaaagtgtaaataaagacttgtgtagttcacgataatcacatccttaatgattaatt
Tuescha-9       tgtgaaagtgtatataaagacttgtgtagttcacgataatcacatccttaatgattaatt
Ha_0            tgtgaaagtgtaaataaagacttgtgtagttcacgataatcacatccttaatgattaatt
Gr_1            tgtgaaagtgtaaataaagacttgtgtagttcacgataatcacatccttaatgattaatt
Vie-0           tgtgaaagtgtatataaagacttgtgtagttcacgataatcacatccttaatgattaatt
                ************ ************************************** ********

Uk_1            aataatgagatcgaatcccttgacttcactcaaacaccttcatgatgatgtcatctgatg
Kondara         aataatgagatcgaatcccttgacttcactcaaacaccttcacgatgatgtcatctgatg
Xan-1           aataatgagatcgaatcccttgacttcactcaaacaccttcacgatgatgtcatctgatg
Van_0           aataatgagatcgaatcccttgacttcactcaaacaccttcacgatgatgtcatctgatg
Ta_0            aataatgagatcgaatcccttgacttcactcaaacaccttcacgatgatgtcatctgatg
Ty_0            aataatgagatcgaatcccttgacttcactcaaacaccttcacgatgatgtcatctgatg
Gel_1           aataatgagatcgaatcccttgacttcactcaaacaccttcacgatgatgtcatctgatg
Hh_0            aataatgagatcgaatcccttgacttcactcaaacaccttcacgatgatgtcatctgatg
Kl-5            aataatgagatcgaatcccttgacttcactcaaacaccttcacgatgatgtcatctgatg
Kro_0           aataatgagatcgaatcccttgacttcactcaaacaccttcacgatgatgtcatctgatg
Jm_0            aataatgagatcgaatcccttgacttcactcaaacaccttcacgatgatgtcatctgatg
Zdr_1           aataatgagatcgaatcccttgacttcactcaaacaccttcacgatgatgtcatctgatg
Lm-2            aataatgagatcgaatcccttgacttcactcaaacaccttcacgatgatgtcatctgatg
```

```
Mc_0          aataatgagatcgaatcccttgacttcactcaaacaccttcacgatgatgtcatctgatg
Tuescha-9     aataatgagatcgaatcccttgacttcactcaaacaccttcacgatgatgtcatctgatg
Ha_0          aataatgagatcgaatcccttgacttcactcaaacaccttcacgatgatgtcatctgatg
Gr_1          aataatgagatcgaatcccttgacttcactcaaacaccttcacgatgatgtcatctgatg
Vie-0         aataatgagatcgaatcccttgacttcactcaaacaccttcacgatgatgtcatctgatg
Ts_1          aataatgagatcgaatcccttgacttcactcaaacaccttcacgatgatgtcatctgatg
              ****************************************** *****************

Uk_1          taacaaaagatattagttaatacttacacgtttatataca--------------------
Kondara       taacaaaagatattagttaatacttacacgttttcatacatatatg-aacttatatgtgt
Xan-1         taacaaaagatattagttaatacttacacgtttacatacatatatgtaa-ttatatgtgt
Van_0         taacaaaagatattagttaatacttacacgtttatatacatatatgtaacttatatgtgt
Ta_0          taacaaaagatattagttaatacttacacgtttacatacatatatgtaacttatatgtgt
Ty_0          taacaaaagatattagttaatacttacacgtttacatacatatatgt--------tgtgt
Gel_1         taacaaaagatattagttaatacttacacgtttatatacatatatgta------------
Hh_0          taacaaaagatattagttaatacttacacgtttacatacatatatgtaacatatatgtgt
Kl-5          taacaaaagatattagttaatacttacacgtttacatacatatatgtaacatatatgtgt
Kro_0         taacaaaagatattagttaatacttacacgtttatatacatatatgtaacttatatgtgt
Jm_0          taacaaaagatattagttaatacttacacgtttatatacatatatgtaacttatatgtgt
Zdr_1         taacaaaagatattagttaatacttacacgtttatatacatatatgtaacttatatgtgt
Lm-2          taacaaaagatattagttaatacttacacgtttatatacatatatgtaacttatatgtgt
Mc_0          taacaaaagatattagttaatacttacacgtttatatacatatatgtaacttatatgtgt
Tuescha-9     taacaaaagatattagttaattcttacacgtttacatacatata----------------
Ha_0          taacaaaagatattagttaatacttacacgtttatatacatatatgtaacttatatgtgt
Gr_1          taacaaaagatattagttaatacttacacgtttatatacatatatgtaacttatatgtgt
Vie-0         taacaaaagatattagttaatacttacacgtttata----------taacttatatgtgt
                   ********************* ***********   *

Uk_1          --------------------------------------------------taaattccac
Kondara       atatatacgtgtaaaacatgagattctcttttgcgatgtcttcatctatataaattccac
Xan-1         atatatacgtgtaaaacatgagattctcttttgcgatgtcttcatctatataaattccac
Van_0         atatatacgtgtaaaacatgagattctcttttgcgatgtcttcatctatataaattccac
Ta_0          atatatacgtgtaaaacatgagattctcttttgcgatgtcttcatctatataaattccac
Ty_0          atatatacgtgtaaaacatgagattctcttttgcgatgtcttcatctatataaattccac
Gel_1         ------------------------------------------------------------
Hh_0          atatatacgtgtaaaacatgagattctcttttgcgatgtcttcatctatataaattccac
Kl-5          atatatacgtgtaaaacatgagattctcttttgcgatgtcttcatctatataaattccac
Kro_0         atatatacgtgtaaaacatgagattctcttttgcgatgtcttcatctatataaattccac
Jm_0          atatatacgtgtaaaacatgagattctcttttgcgatgtcttcatctatataaattccac
Zdr_1         atatatacgtgtaaaacatgagattctcttttgcgatgtcttcatctatataaattccac
Lm-2          atatatacgtgtaaaacatgagattctcttttgcgatgtcttcatctatataaattccac
Mc_0          atatatacgtgtaaaacatgagattctcttttgcgatgtcttcatctatataaattccac
Tuescha-9     ---tatacgtgtaaaacatgagattctcttttgcgatgtcttcatctatataaattccac
Ha_0          atatatacgtgtaaaacatgagattctcttttgcgatgtcttcatctatataaattccac
Gr_1          atatatacgtgtaaaacatgagattctcttttgcgatgtcttcatctatataaattccac
Vie-0         atatatacgtgtaaaacatgagattctcttttgcgatgtcttcatctatataaattccac


Uk_1          tttttcctttagctgtggatcttctttttttctaccgtattctatatttttccttacttt
Kondara       tttttcctttagctgtagatcttctttttttctaccgtactctatatttttccttacttt
Xan-1         tttttcctttagttgt-g-tcttctttttttctatcgtattctatatttttccttacttt
Van_0         tttttcctttagctgtggatcttctttttttctaccgtattctatatttttccttacttt
Ta_0          tttttcctttagctgtggatcttctttttttctaccgtattctatatttttccttacttt
Ty_0          tttttcctttagctgtggatcttctttttttctaccgtactctatatttttccttacttt
Gel_1         ----------------------------tttctaccgtattctatatttttccttacttt
Hh_0          tttttcctttagctgtggatcttctttttttctaccgtactctatatttttccttacttt
Kl-5          tttttcctttagctgtggatcttctttttttctaccgtactctatatttttccttacttt
Kro_0         tttttcctttagctgtggatcttctttttttctaccgtactctatatttttccttacttt
Jm_0          tttttcctttagctgtggatcttctttttttctaccgtactctatatttttccttacttt
Zdr_1         tttttcctttagctgtggatcttctttttttctaccgtactctatatttttccttacttt
Lm-2          tttttcctttagctgtggatcttctttttttctaccgtactctatatttttccttacttt
Mc_0          tttttcctttagctgtggatcttctttttttctaccgtactctatatttttccttacttt
Tuescha-9     tttttcctttagctgtggatcttctttttttctaccgtactctatatttttccttacttt
```

```
Ha_0           ttttttcctttagctgtggatcttctttttttctaccgtactctatatttttccttacttt
Gr_1           ttttttcctttagctgtggatcttctttttttctaccgtactctatatttttccttacttt
Vie-0          ttttttcctttagctgtggatcttctttttttctatcgtattctatatttttccttacttt
                     ******  *** *********************

       Uk_1           cgtggcaaaaactttacgatctttcaATGTCAGAAGAATTTCAAGA
       Kondara        cgtggtaaaaactttacgatctttcaATGTCAGAAGAATTTCAAGA
       Xan-1          cgtggcaaaaactttacgatctttcaATGTCAGAAGAATTTCAAGA
       Van_0          cgtggcaaaaactttacgatctttcaATGTCAGAAGAATTTCAAGA
       Ta_0           cgtggcaaaaactttacgatctttcaATGTCAGAAGAATTTCAAGA
       Ty_0           ctaggcaaaaactttacgatctttcaATGTCAGAAGAATTTCAAGA
       Gel_1          cgtggcaaaaactttacgatctttcaATGTCAGAAGAATTTCAAGA
       Hh_0           ctaggcaaaaactttacgatctttcaATGTCAGAAGAATTTCAAGA
       Kl-5           ctaggcaaaaactttacgatctttcaATGTCAGAAGAATTTCAAGA
       Kro_0          ctaggcaaaaactttacgatctttcaATGTCAGAAGAATTTCAAGA
       Jm_0           ctaggcaaaaactttacgatctttcaATGTCAGAAGAATTTCAAGA
       Zdr_1          ctaggcaaaaactttacgatctttcaATGTCAGAAGAATTTCAAGA
       Lm-2           ctaggcaaaaactttacgatctttcaATGTCAGAAGAATTTCAAGA
       Mc_0           ctaggcaaaaactttacgatctttcaATGTCAGAAGAATTTCAAGA
       Tuescha-9      ctaggcaaaaactttacgatctttcaATGTCAGAAGAATTTCAAGA
       Ha_0           ctaggcaaaaactttacgatctttcaATGTCAGAAGAATTTCAAGA
       Gr_1           ctaggcaaaaactttacgatctttcaATGTCAGAAGAATTTCAAGA
       Vie-0          cgtggcaaaaactttacgatctttcaATGTCAGAAGAATTTCAAGA
                      *  ** ****************************************
```

Fig. S5. Promoter analysis of *AtS40-3* and *PPR* genes. 1500 bp promoter region indicating major deletion (red coloured) in the putative bi-directional promoter of *AtS40-3* and *PPR* among lowly susceptible accessions.

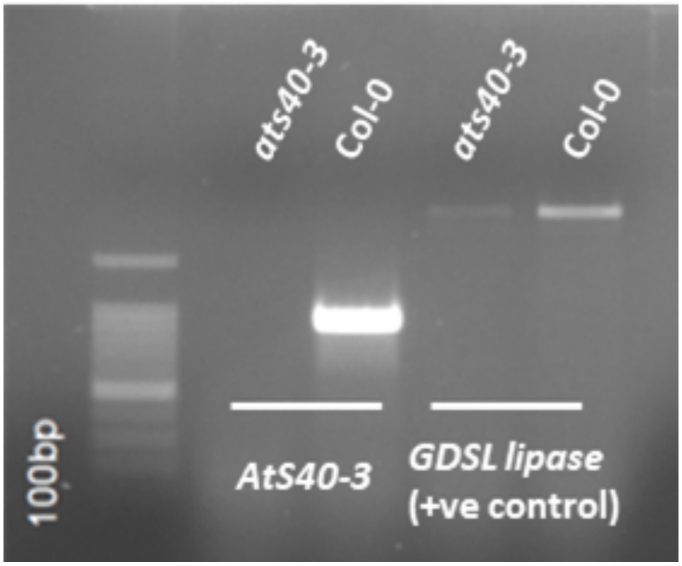

Fig. S6: RT-PCR for presence or absence of *AtS40-3* expression in Col-0 or *ats40-3* mutant. GDSL lipase was used as positive control.

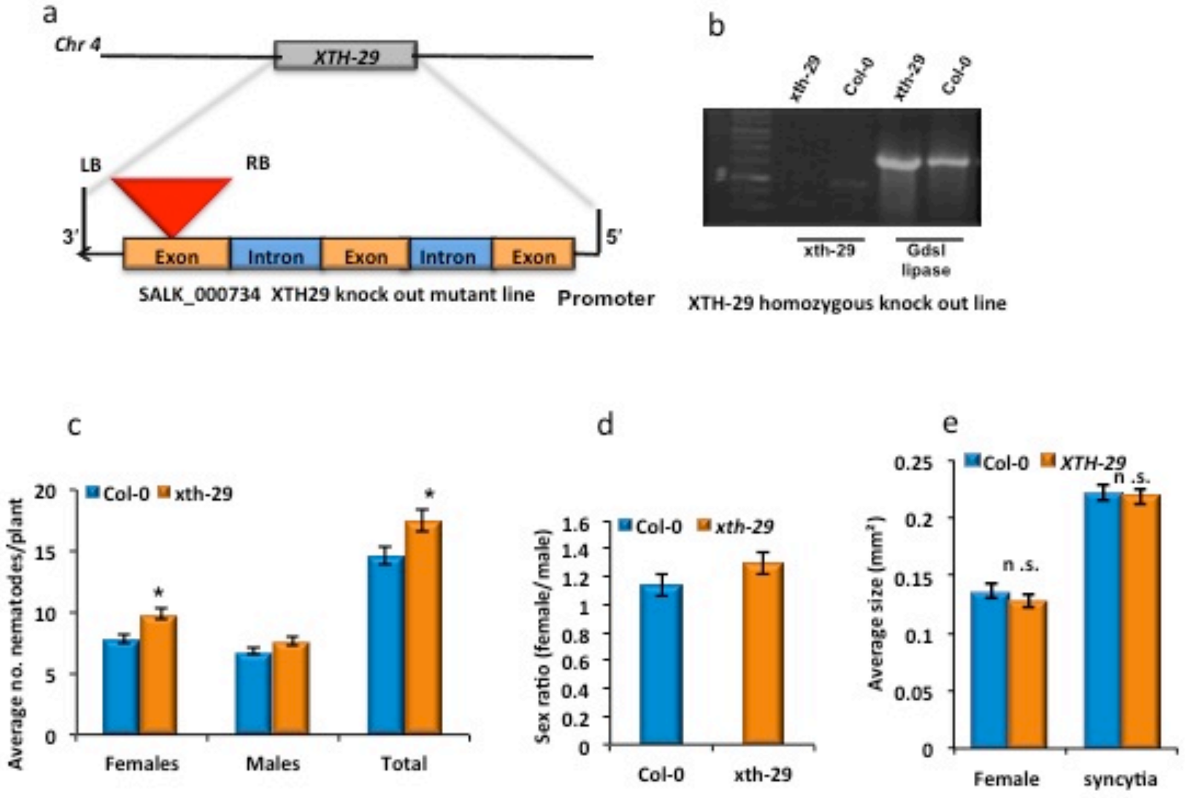

Fig. S7: Cyst nematode infection assays in XTH-29mutant plants. (a) Schematic diagram of a T-DNA line (SALK_000734) showing insertion in exon of XTH-29. (b) RT-PCR for presence or absence of GDSL expression in Col-0 or *xth-29* mutant. (c) Average number of female and male nematodes per plant present in Col-0 compared to *xth-29* mutants compared at 12 dpi. (d) Average size of females and associated syncytia in Col-0 compared to *xth-29* at 14 dpi. Bars represent mean and standard error of three independent experiments. Data was analyzed for the significance difference using T-test ($p<0.05$). n.s., not significant.

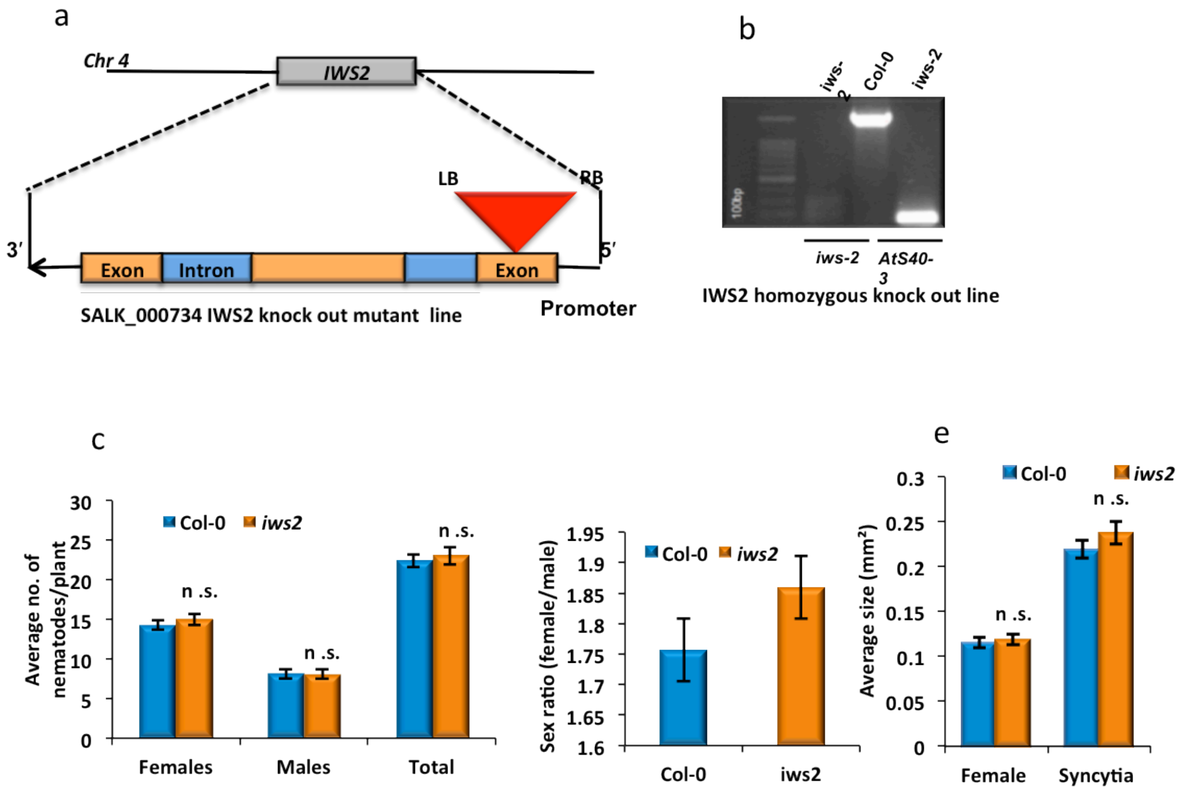

Fig. S8: Cyst nematode infection assays in IWS2mutant plants. (a) Schematic diagram of a T-DNA line (SALK_149853C) showing insertion in exon of **IWS2**. (b) RT-PCR for presence or absence of GDSL expression in Col-0 or *xth-29* mutant. (c) Average number of female and male nematodes per plant present in Col-0 compared to *iws2* mutants compared at 12 dpi. (d) Average size of females and associated syncytia in Col-0 compared to *iws2* at 14 dpi. Bars represent mean and standard error of three independent experiments. Data was analyzed for the significance difference using T-test ($p<0.05$). n.s., not significant.
